# Laminarinase from *Flavobacterium* sp. reveals the structural basis of thermostability and substrate specificity

**DOI:** 10.1038/s41598-017-11542-0

**Published:** 2017-09-12

**Authors:** Hui-Min Qin, Takuya Miyakawa, Akira Inoue, Akira Nakamura, Ryuji Nishiyama, Takao Ojima, Masaru Tanokura

**Affiliations:** 1 Laboratory of Basic Science on Healthy Longevity, Department of Applied Biological Chemistry, Graduate School of Agricultural and Life Sciences, The University of Tokyo, 1-1-1 Yayoi, Bunkyo-ku, Tokyo, 113-8657 Japan; 2 College of Biotechnology, Tianjin University of Science and Technology, No. 29, 13th Avenue, Tianjin, 300457 China; 3 Laboratory of Marine Biotechnology and Microbiology, Graduate School of Fisheries Sciences, Hokkaido University, 3-1-1 Minato-cho, Hakodate, 041-8611 Japan

## Abstract

Laminarinase from *Flavobacterium* sp. strain UMI-01, a new member of the glycosyl hydrolase 16 family of a marine bacterium associated with seaweeds, mainly degrades β-1,3-glucosyl linkages of β-glucan (such as laminarin) through the hydrolysis of glycosidic bonds. We determined the crystal structure of ULam111 at 1.60-Å resolution to understand the structural basis for its thermostability and substrate specificity. A calcium-binding motif located on the opposite side of the β-sheet from catalytic cleft increased its degrading activity and thermostability. The disulfide bridge Cys31-Cys34, located on the β2-β3 loop near the substrate-binding site, is responsible for the thermostability of ULam111. The substrates of β-1,3-linked laminarin and β-1,3-1,4-linked glucan bound to the catalytic cleft in a completely different mode at subsite -3. Asn33 and Trp113, together with Phe212, formed hydrogen bonds with preferred substrates to degrade β-1,3-linked laminarin based on the structural comparisons. Our structural information provides new insights concerning thermostability and substrate recognition that will enable the design of industrial biocatalysts.

## Introduction

Glucans with β-1,3-linkages are widely distributed in nature and are found in bacteria, fungi, plants, and algae. β-1,3-linked glucan is the main constituent of botanical and fungal cell walls; it is a major structural polysaccharide^1^. Laminarin is a storage polysaccharide of marine macroalga^1, 2^. Its main chain consists of glucose with β-1,3-linkages and partial branches connected through β-1,6-linkages. Ratios of β-1,3- and β-1,6-linkages are diverse, *e*.*g*., 7:1 in *Laminaria digitata* and *L*. *hyperborea* ^3^ and 3:2 in *Eisenia bicyclis* ^4^. Laminarin has shown anti-apoptotic and anti-tumor activities^5, 6^. Laminarin is also a potential source of fermentable sugars for bioethanol production^7^. Therefore, it has received attention in the design of biocatalysts for the development of a cost-competitive process for converting laminarin into fermentable sugar for the widespread utilization^8, 9^. Some other extracellular polysaccharides, such as curdlan and lichenin, have also been demonstrated to be non-toxic, and have applications in the food and pharmaceutical industries^10^. Curdlan is unbranched and consists of glucosyl residues that are linked by β-D-1,3 bonds with its degree of polymerization being about 135 glucose residues^11^. Lichenin could form symbiotic relationships with algae in lichens has a ratio of β-D-1,4-glucopyranosyl to β-D-1,3-glucopyranosyl residues of 2.3:1 in *Cetraria islandica* ^12^.

Laminarinase (EC 3.2.1.6, known as β-1,3-glucanohydrolase or β-1,3-glucanase) catalyzes the hydrolysis of 1,3- and 1,4-linkages in β-D-glucans^13^. Laminarinase can play various physiological roles in bacteria, viruses, and plants^14–16^. In bacteria, laminarinases are classified into exo-β-1,3-glucanases (EC 3.2.1.58) and endo-β-1,3-glucanases (laminarinase; EC 3.2.1.6 and EC 3.2.1.39), which share high sequence similarity to endo-β-1,3-1,4-glucanases (EC 3.2.1.73). Exo-/endo-β-1,3-glucanases hydrolyze β-1,3-glucans such as laminarin and release glucose residues or oligosaccharides, while endo-β-1,3-1,4-glucanases may also hydrolyze polysaccharides containing β-1,4 linkages, such as lichenin^17^. Therefore, both laminarinases and β-1,3-1,4-glucanases are expected to become promising biocatalysts in the generation of biochemicals and bioenergy^18, 19^.

The Carbohydrate-Active Enzymes database (CAZy; http://www.cazy.org) provides a sequence-based family classification linking the sequence to the specificity and 3D structure of the enzymes^20^, in which most bacterial laminarinases and β-1,3-1,4-glucanases belong to the GH16 family. Some members of this family have been characterized, and crystal structures have been analyzed for enzymes from *Thermotoga maritima* ^21^, hyperthermophile *Pyrococcus furiosus* ^17, 22^, *Nocardiopsis* sp. strain F96^23^, and *Zobellia galactanivorans* ^24^. It is not easy to classify GH16 family enzymes aside from a classical sandwich-like β-jelly roll fold composed of two antiparallel β-sheets packed against each other^25, 26^. Determining the crystal structures of laminarinases/glucanases provides useful examples of versatile yet specific protein-carbohydrate interactions, which could not be precisely predicted by sequence alignment alone.

*Flavobacterium* sp. UMI-01 is a novel bacterium recently identified from decayed brown algae; it has been grown in medium containing either alginate or laminarin as a sole carbon source^27^. *Flavobacteria* contains a high abundance of family GH16 laminarinases, underlying the environmental importance of decomposing algal biomass. Genomic sequence analysis revealed the presence of a candidate gene for GH16 β-1,3-laminarinase, which was designated as ULam111. This enzyme consists of 235 residues and has a molecular mass of approximately 27 kDa. Herein, we determined the crystal structure of ULam111 at 1.60-Å resolution. The structure revealed new insights concerning the thermostability and substrate recognition for the degradation of polysaccharides by the laminarinase family, which may be suitable for beer brewing and feed additives.

## Results and Discussion

### Overall structure of ULam111

The crystal structure of ULam111 was refined to 1.60-Å resolution. Two protein molecules and 670 water molecules were observed in the asymmetric unit of the ULam111 crystal. The *R* and *R*_free_ values of the final model were 17.6 and 20.6%, respectively. In the Ramachandran plot, 97.4% of residues were included in the favored region, and 2.6% were in the allowed region. The refinement statistics are summarized in Table 1.

**Table 1:** Data Collection and Refinement Statistics.

	Se-labeled	Native
**Data Collection**		
Beamline	PF BL-5A	PF BL-5A
Wavelength (Å)	0.97935	1.00000
Space group	*P*2_1_	*P*2_1_2_1_2_1_
Unit-cell parameters (Å)	*a* = 66.6, *b* = 64.5, *c* = 70.4	*a* = 64.6, *b* = 80.9, *c* = 109.5
(°)	*β* = 106.5	
Resolution (Å)^a^	50.0−2.50 (2.56−2.50)	50.0−1.60 (1.64−1.60)
No. of unique reflections	38794	76300
Completeness (%)^a^	99.8 (99.7)	99.9 (100.0)
*R*_sym_^a,b^	0.081 (0.128)	0.053 (0.305)
〈*I*/σ(*I*)〉^a^	19.80 (13.24)	25.71 (6.60)
Redundancy	7.4	7.2
**Refinement**		
*R*_work_/*R*_free_(%)		17.6/20.6
No. of atoms		
Protein		3753
Water		670
RMSD^c^		
Bond length (Å)		0.008
Bond angle (°)		1.180
Ramachandran plot (%)		
Favored		97.4
Allowed		2.6
Disallowed		0
PDB code		5WUT

^a^Values in parentheses are for the highest resolution shell.

^b^ *R*_sym_ = Σ_*hkl*_ [(Σ_*i*_ |*I*_*i*_ − 〈*I*〉|)/Σ_*i*_ *I*_*i*_], where *I*_*i*_ is the *i*th intensity measurement of the reflection *hkl*, including symmetry-related reflections, and 〈*I*〉 is its average.

^c^RMSD, root-mean-square deviation.

**Figure 1:**
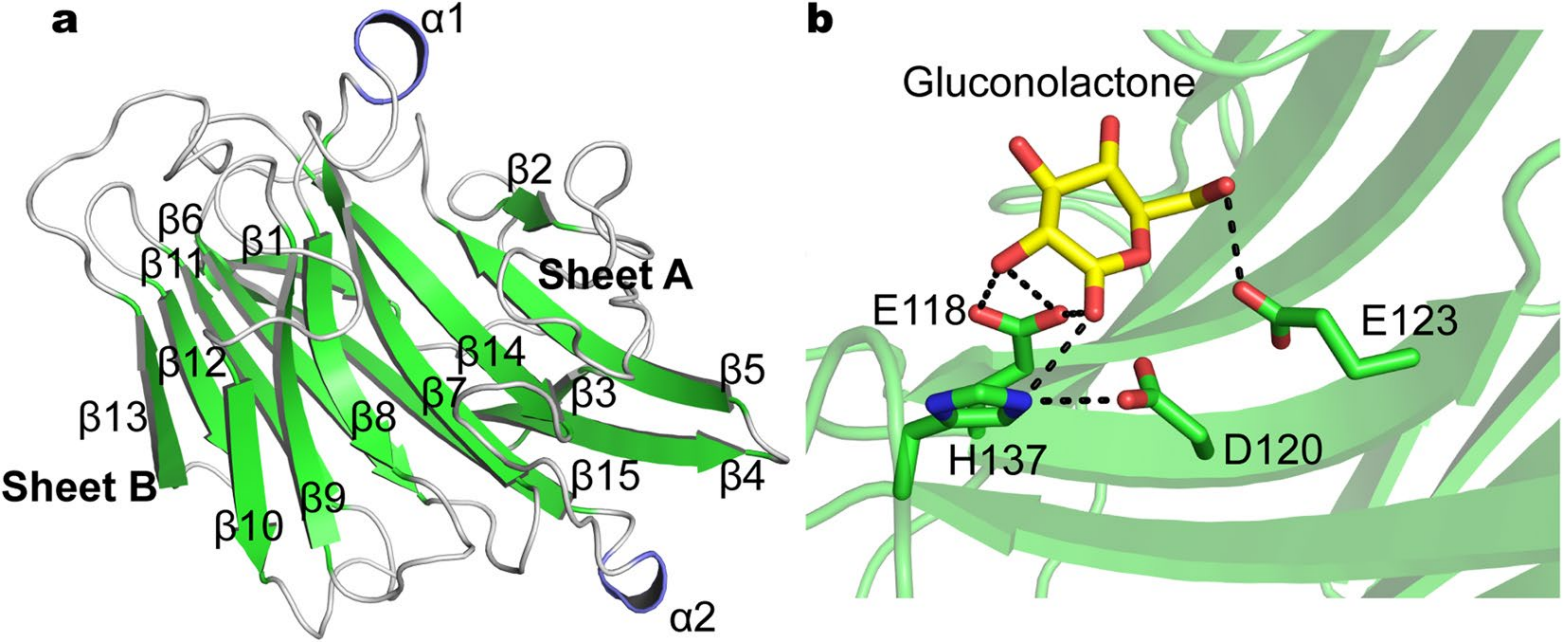
(**a**) Ribbon representation of the overall ULam111 structure. The β-strands of the β-jelly roll fold are shown in green. The α-helices are shown in deep blue. The loops are white. (**b**) Active site residues in ULam111 structure. Gluconolactone (in yellow sticks) was modeled on the superposition of TmLam from *Thermotoga maritima* (PDB code: 3AZX).

The overall structure of ULam111 contained two α-helices and 15 β-strands and possessed a β-jelly roll fold (Fig. 1a) that is adopted in GH16 family enzymes^17, 21–24^. The fold comprises two antiparallel β-sheets (Sheet A and Sheet B). Sheet A consists of seven β-strands (β2, residues 24–26; β5, residues 66–73; β7, residues 97–105; β8, residues 118–124; β9, residues 131–137; β10, residues 148–152; and β14, residues 200–208), and sheet B consists of eight β-strands (β1, residues 6–11; β3, residues 49–52; β4, residues 55–63; β6, residues 83–90; β11, residues 161–167; β12, residues 172–176; β13, residues 179–184; and β15, residues 224–235).

### Characteristics of the active site

The catalytic residues included Glu118, Asp120, Glu123, and His137 (Fig. 1b). On the basis of the conserved catalytic mechanism^21, 28^, Glu118 was assumed to be the nucleophile that directly attacks C1 of the sugar ring, and Glu123 was hypothesized to function as the proton donor. The interaction of Asp120 with His137 via a hydrogen bond was considered to stabilize the substrate.

### Comparison of the ULam111 structure with other family enzymes

A structure homology search using Dali^29^ showed that the overall structure of ULam111 is highly similar to previously reported enzymes, including TmLam from *Thermotoga maritima* (PDB code, 3AZZ; *Z*-score, 34.8; r.m.s.d., 1.5 Å; sequence identity, 45%), BglF from *Nocardiopsis* sp. strain F96 (PDB code, 2HYK; *Z*-score, 33.8; r.m.s.d., 1.7 Å; sequence identity, 44%) and PfLamA from the hyperthermophile *Pyrococcus furiosus* (PDB code, 2VY0; *Z*-score, 33.8; r.m.s.d., 1.4 Å; sequence identity, 43%).

**Figure 2:**
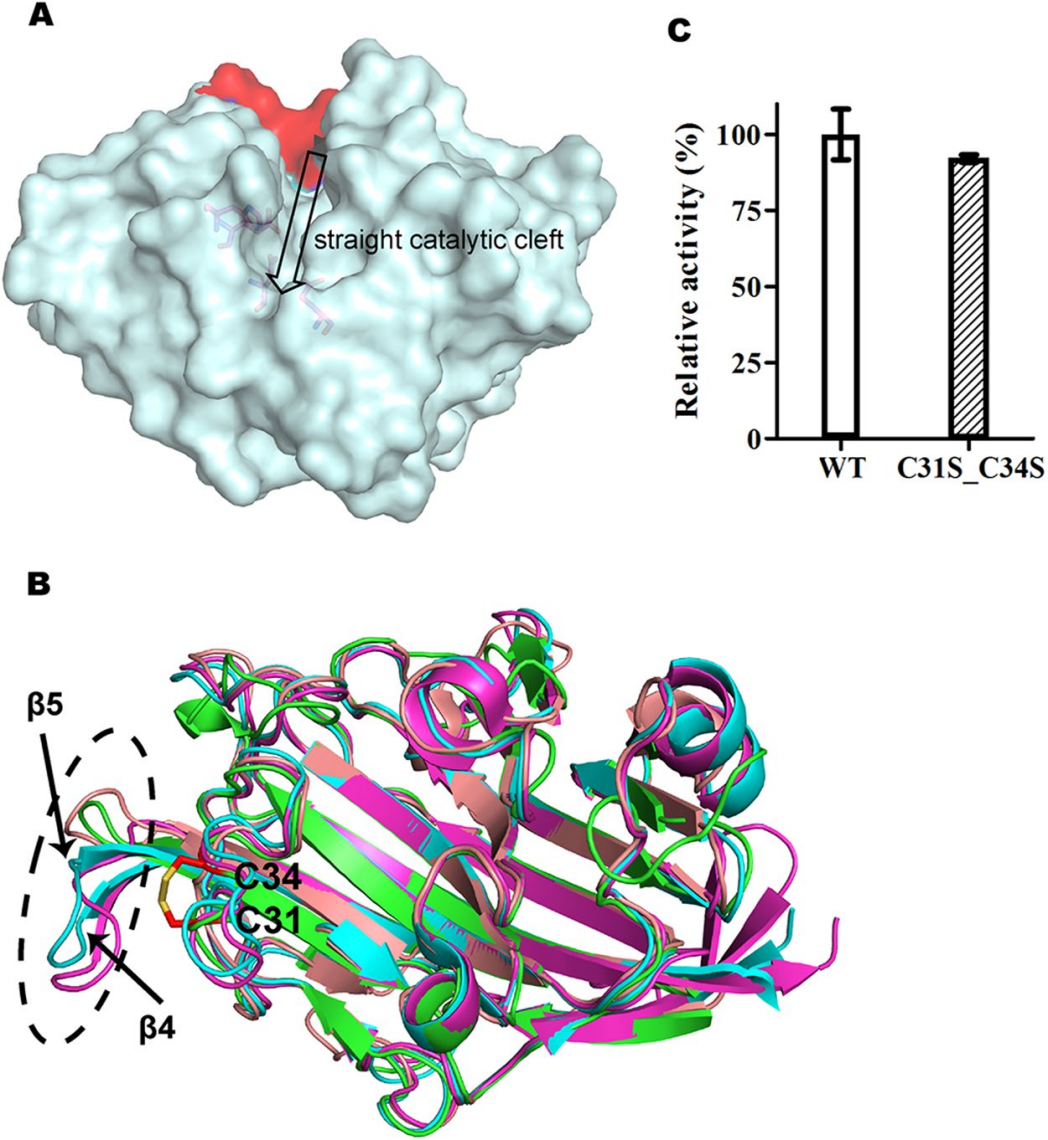
(**a**) The surface of ULam111 displaying a straight topology. The additional loop of ZgLamA is red. (**b**) Superposition of the structures of ULam111 (green), BglF from *Nocardiopsis* sp. strain F96 (salmon), TmLam (cyan), and PfLamA from hyperthermophile *Pyrococcus furiosus* (magenta). The disulfide linkage of Cys31-Cys34 is shown as red sticks. The black dashed cycles showed different structures between ULam111 and other GH16 family enzymes. (**c**) Relative activity of ULam111 mutant toward laminarin. The activity of wild-type ULam111 is represented as 100 and the error bars are standard deviations (*n* = 3).

Although ULam111 shared >43% sequence identity with TmLam, BglF and PfLamA, structural determination by molecular replacement using a structure model of TmLam was not successful, indicating that there may be conformational differences between ULam111 and some family enzymes. The superposition of ULam111 with TmLam, BglF, and PfLamA showed that these proteins contained the common β-jelly roll folds and a straight groove for substrate binding (Fig. 2). ULam111 shared the bulging loop of β2-β3 with TmLam and PfLamA. The comparison of ULam111 and ZgLamA showed that ULam111 lacked the flexible loop at the entrance to regulate the recognition of β-1,3-1,4-linked substrates. However, the disulfide bond of Cys31-Cys34 only existed in ULam111 (Figs 2b and S1). This loop may partly regulate substrate recognition, as it was located above the substrate-binding site and slightly decreased the degrading activity compared with the wild-type enzyme (Fig. 2c). The β-strands of β4 and β5 exhibiting a different spatial conformation were considered to serve as a structural feature because they were on the opposite side of the catalytic cleft.

### Comparison of substrate binding site

**Figure 3:**
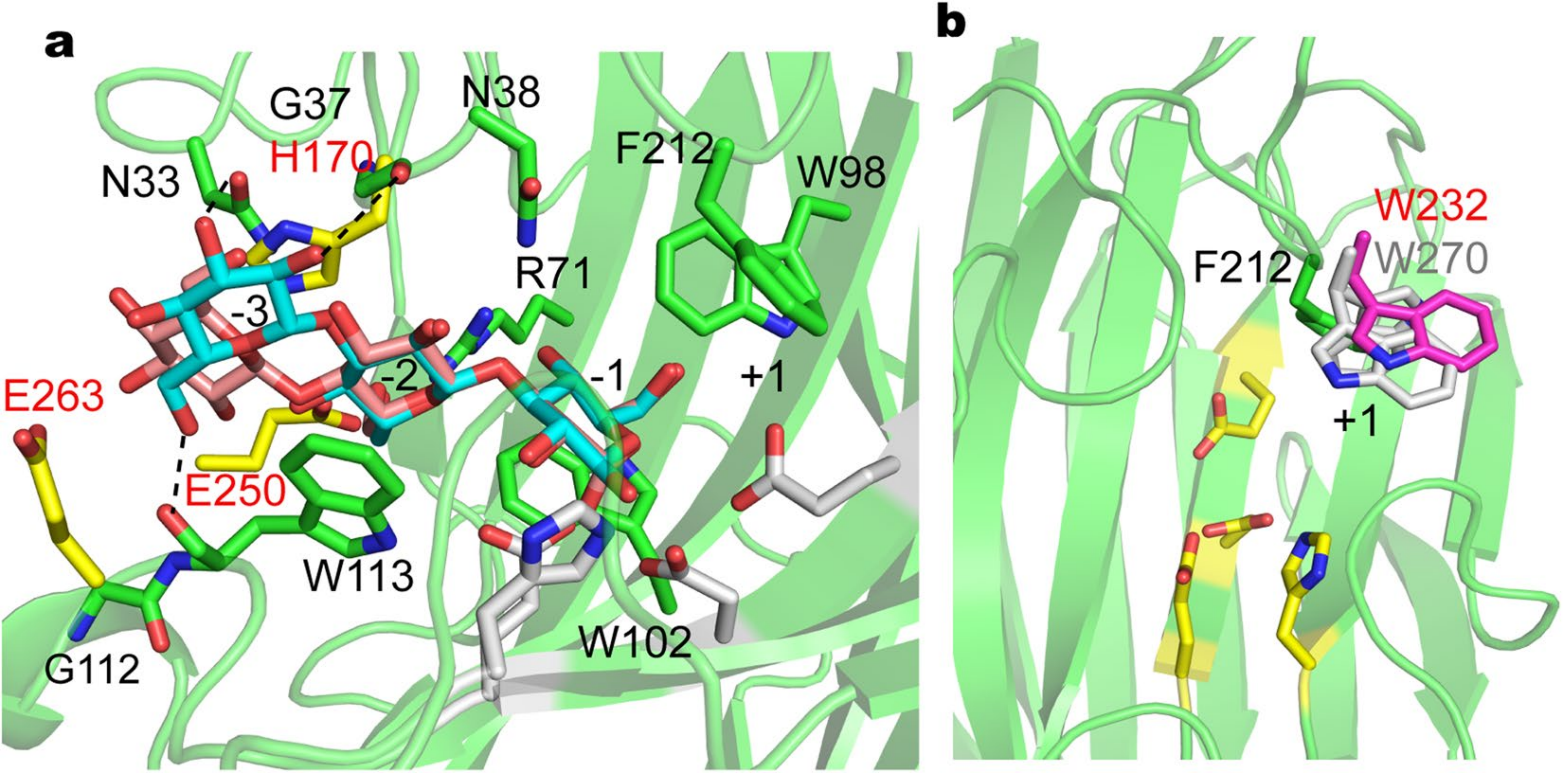
(**a**) Molecular basis for the recognition of β-1,3-linked laminarin and β-1,3-1,4-linked glucans by ULam111. The β-1,3-linked laminarin and β-1,3-1,4-linked glucans are cyan and salmon colored, respectively. The amino acids in ZgLamA are yellow. (**b**) Comparison of spatial orientation of Phe212 (green) in ULam, Trp270 (white) in PfLamA, and Trp232 (magenta) in TmLam.

Some β-strands of Sheet A twisted somewhat to form an electronegative-rich cleft where the substrates were implicated to bind (Fig. S2). It is obvious that ULam111 binds β-1,3-linked or β-1,3-1,4-linked substrates in a straight catalytic cleft and cleaves the glucosidic bond in an open form because there are no additional loops covering the active site (Fig. 2a). The sugar ring may form hydrogen bonds at the negatively charged binding site of ULam111 with hydrophilic residues, such as Asn33, Gly37, Asn38, and Arg71, and hydrophobic-stacking interactions with Trp98, Trp102, and Trp113, whereas most of the residues are conserved in the GH16 family enzymes (Fig. 3a). Notably, Phe212, located above the catalytic residues of positive substrate-binding site + 1, was not conserved (tryptophan in GH16 family enzymes) and showed different spatial orientations than other enzymes (Fig. 3b). It was reported to regulate substrate recognition in the release of glucose in carbohydrate hydrolysis^21^.

### Thermostability of ULam111

**Figure 4:**
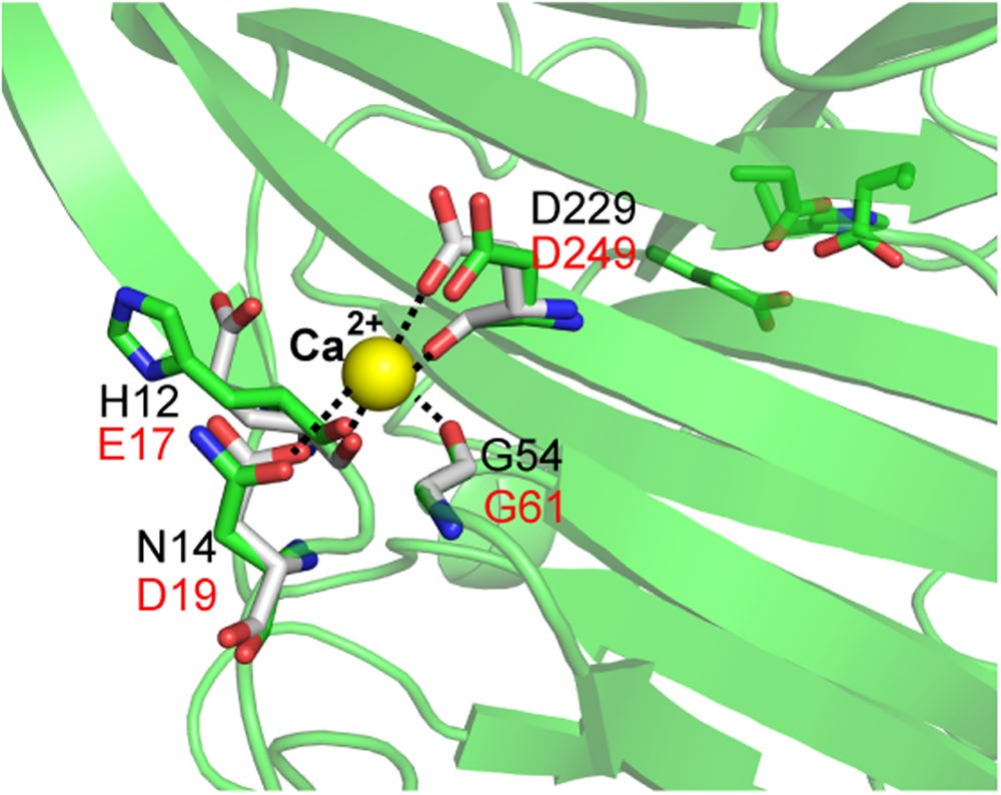
Comparison of calcium binding motif. The amino acids are colored in green (ULam111), and white (TmLam), respectively. Calcium is displayed as yellow sphere.

**Figure 5:**
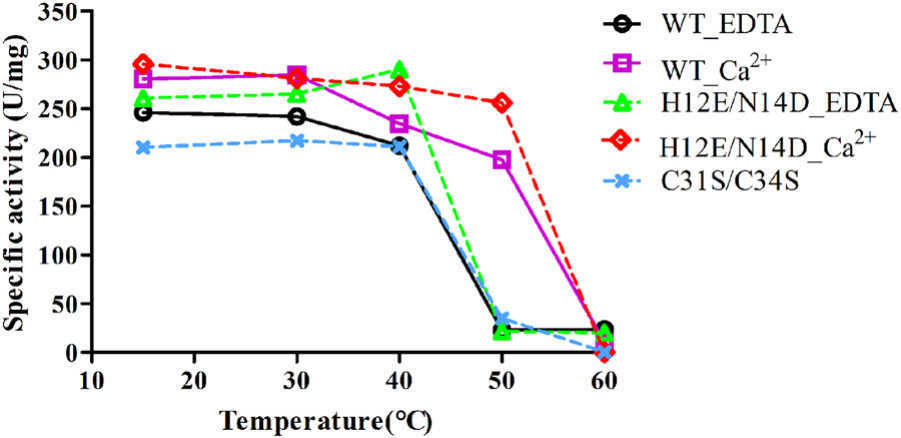
The thermostability of ULam111 and its mutants. Results are presented as the mean value of three independent experiments.

ULam111 contained a special calcium-binding motif on the opposite side of the β-sheet from the catalytic cleft (Fig. 4), which was composed of His12, Asn14, Gly54, and Asp229. The calcium ion was coordinated to the carbonyl and carboxylate O atoms of Asp229, the carbonyl O atom of Gly54, and the carbonyl O atom of Asn14. This motif is known to increase thermostability in GH16 family enzymes^30^. However, the calcium-binding motif is not conserved in this family. The residues of Asn14 and His12 in ULam111 were positioned near glutamic acid and aspartic acid in other enzymes (Fig. 4). The H12E/N14D substitution could increase the degradative activity toward laminarin and thermostability from 15 °C to 40 °C (Fig. 5). It is interesting that the calcium ion could significantly increase the activity and thermostability of wild-type and mutant enzymes. Especially at 50 °C, the enzymes retained approximately 80% activity under 1.0 mM CaCl_2_. Therefore, both the mutant and calcium ion were required to enhance thermostability.

ULam111 formed one intramolecular disulfide bond at Cys31-Cys34 in the loop between β2 and β3, which was considered to serve as a key structural feature in stabilizing the loop above the catalytic cleft of ULam111. At the same position, TmLam has the histidine and proline residues, while PfLamA adopts the isoleucine and proline residues (Fig. S1). The C31S/C34S mutant showed decreased thermostability in our detectable temperature range (especially from 15 to 30 °C). Therefore, the loop including Cys31-Cys34 played a pivotal role in regulating thermostability.

### Substrate specificity of ULam111

**Figure 6:**
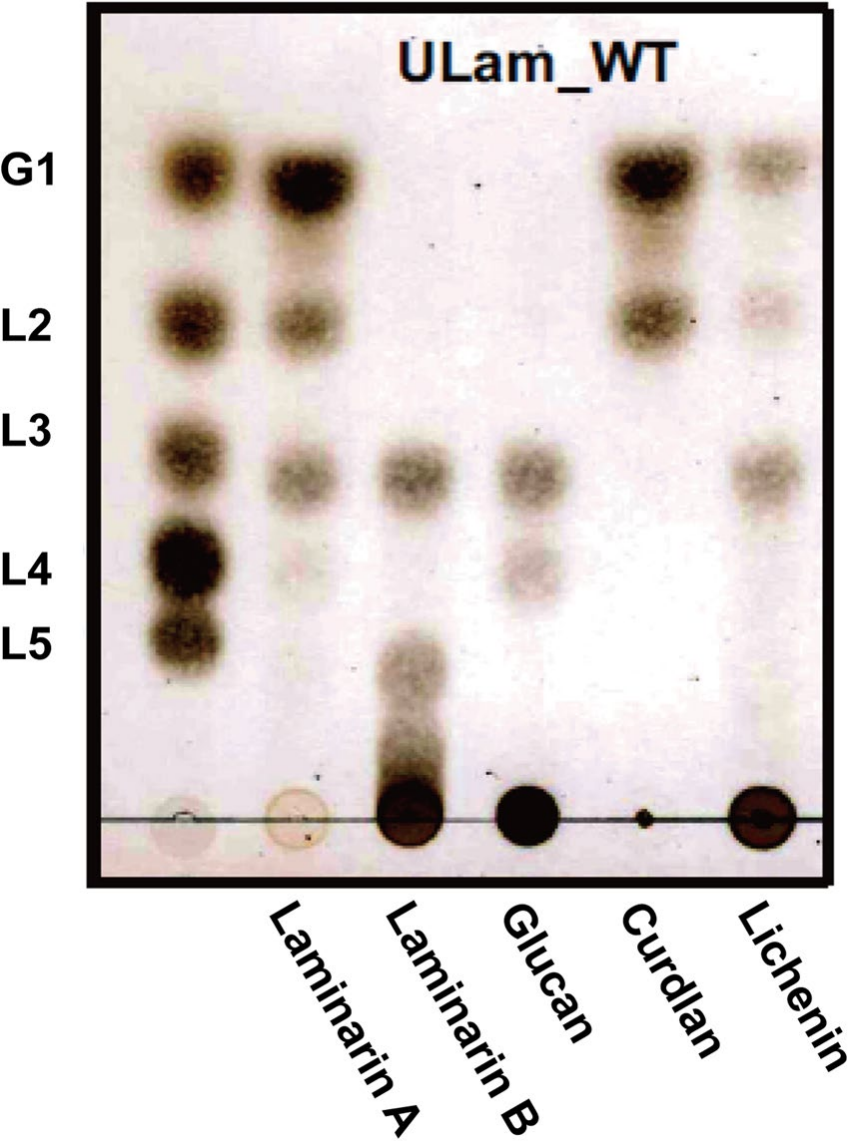
Hydrolysis of laminarin (A, β-1,3-1,6-linkages from *Laminaria digitata* and B, β-1,3-1,6-linkages from *Eisenia bicyclis*), glucan (β-1,3-1,6-linkages from *Flammulina velutipes*) curdlan (β-1,3-linkages from *Alcaligenes faecalis*), and lichenin (β-1,3-1,4-linkages from *Cetraria islandica*) by ULam111 was monitored by TLC. G1, glucose; L2, laminaribiose; L3, laminaritriose; L4, laminaritetraose; and L5, laminaripentaose. The cropped blots were displayed and the original image was provided in supplementary information.

The kinetic parameters of ULam111 on different substrates are summarized in Table 2. ULam111 are characterized by *K*_m_ values of between 0.56 and 1.60 mg ml^−1^ and catalytic efficiencies of between 2.9 and 58 ml mg^−1^ s^−1^. ULam111 showed the high activity toward laminarin A from *Laminaria digitata* and curdlan from *Alcaligenes faecalis*. Curdlan is composed only β-1,3-glycosidic bonds, and the content of β-1,3-linkage in laminarin A is higher than those in laminarin B and glucan from *Eisenia bicyclis* and *Flammulina velutipes*, respectively. In addition, *k*_cat_ value toward lichenin (β-1,3/β-1,4) was 10-fold lower than that toward curdlan (β-1,3) and laminarin A (β-1,3/β-1,6). It was reported that substrate specificity was dependent on a straight groove or bent shape suitable for binding the linear β-1,3-1,4-linkages of glucan and β-1,3-glucan, respectively^24, 31^. In ZgLamA, the unique additional loop including His170, Glu250, and Glu263 on bent groove was attributed to the binding of β-1,3-linkage. The absence of this loop in BglF and deletion in PfLamA both contributed to the substrate selectivity toward β-1,3-1,4-linkages of glucan^16, 23^. However, ULam111 showed a predominant specificity toward the helical conformation of the β-1,3-linkages of laminarin (Fig. 6 and Table 2) with a straight groove at its distinctive mode compared with ZgLamA, BglF and PfLamA. A structural superposition of ULam111 with ZgLamA revealed that ULam111 displayed no additional loop to bind laminarin (Fig. 2). Figure 3 showed that ULam111 bound both substrates with a similar β-1,3-linkage mode at subsites −1 and −2. However, the glucosyl moiety was bound to the catalytic cleft in a completely different mode at subsite −3. Some unique residues in ULam111, such as Asn33 and Trp113, could form hydrogen bonds that prefer to degrade β-1,3-linked laminarin. On the other hand, the different *K*_m_ values were observed between lichenin (β-1,3/β-1,4) and glucan (β-1,3/β-1,6) (Table 2), which showed that the β-1,6-linkage of substrate has a greater influence on *K*_m_ than the β-1,4-linkage. These results suggest that the β-1,6-linkage may have an inhibitory effect against the binding of β-1,3-linkage at the active site because 1,6-linked glucosyl residues on laminarin/glucan constitute branch points on the all-1,3-linked backbone^32^. Therefore, ULam111 showed the high affinity toward β-1,3-linked substrates. However, lichenin is a strictly linear polysaccharide composed of a 1,4-linkage backbone which also contains 1,3-linked “kinks” randomly throughout the chain^32^. It may explain the phenomenon that ULam111 showed low activity toward β-1,4-linked lichenin.

**Table 2:** Kinetic Parameters of ULam111 Wild Type and F212W on Several Substrates.

Enzymes	Substrates	Glycosidic bond(s)	*K*_m_ (mg ml^−1^)	*V*_max_ (U mg^−1^)	*k*_cat_ (s^−1^)	*k*_cat_/*K*_m_ (ml mg^−1^ s^−1^)
WT						
	laminarinA	β-1,3/β-1,6	0.56 ± 0.10	360 ± 19.9	17	31
	laminarinB	β-1,3/β-1,6	1.39 ± 0.22	194 ± 13.7	9.3	6.7
	glucan	β-1,3/β-1,6	1.60 ± 0.30	105 ± 9.0	5.0	3.1
	curdlan	β-1,3	0.30 ± 0.03	366 ± 9.9	18	58
	lichenin	β-1,3/β-1,4	0.65 ± 0.11	39.0 ± 2.2	1.9	2.9
F212W						
	laminarinA	β-1,3/β-1,6	1.22 ± 0.17	214 ± 12.3	10	8.4
	laminarinB	β-1,3/β-1,6	2.18 ± 0.47	154 ± 16.9	7.4	3.4
	glucan	β-1,3/β-1,6	2.72 ± 0.71	101 ± 14.6	4.8	1.8
	curdlan	β-1,3	0.97 ± 0.17	213 ± 14.3	10	11
	lichenin	β-1,3/β-1,4	1.65 ± 0.23	27.2 ± 1.76	1.3	0.8

All assays were repeated three times, and the data are shown as mean ± S.D.

Phe212 (tryptophan in the GH16 family enzymes) showed a different spatial orientation than that of other enzymes (Fig. 3b). Trp232 in TmLam may form hydrophobic interactions with a flexible GASIG loop in the closed form, contributing to regulation of exo-cleavage activity and preferred release of glucose in carbohydrate hydrolysis^21^. Trp270 in PfLamA showed a dual spatial orientation to regulate substrate specificity. To investigate the role of Phe212 in substrate recognition, we mutated Phe212 to tryptophan. The result showed that F212W mutant of ULam111 led to the decreased degrading activity (*V*_max_ and *k*_cat_/*K*_m_) compared with the wild-type enzyme toward all of the tested substrates (Table 2). The decreased ratio of *k*_cat_/*K*_m_ toward curdlan (β-1,3) was the highest among those toward all tested substrates. F212W mutant reduced the degrading activity toward laminarin to glucose within the initial 30 min (Fig. S4), which implied that phenylalanine has less steric hindrance than tryptophan and was the optimal residue for substrate recognition.

## Materials and Methods

### Cloning, expression, and purification

A gene encoding the candidate β-1,3-glucanase, termed ULam111 (GenBank accession no. LC202090), was found in the draft genome sequence of the *Flavobacterium* sp. strain UMI-01 as previously analyzed^27^. Genomic DNA was prepared from strain UMI-01 as previously described^27^ and was used as a template for genomic polymerase chain reaction (PCR) with the Q5 High-Fidelity DNA Polymerase DNA polymerase (New England Biolabs, Ipswich, MA) and a pair of specific primers, F1 (5′-GTTCGGCTAAAACACTCGAAGCTG-3′) and F2 (5′-ATCAAATGCAATCTAAATTCCGTG-3′), for the 5′- and 3′-untranslated regions, respectively. PCR was conducted using temperature settings of 95 °C for 5 min followed by 30 cycles of 95 °C for 15 s, 55 °C for 15 s, and 72 °C for 45 s. The final step for extension was 72 °C for 2 min. Amplified DNA was subcloned into the pTac-1 vector (BioDynamics, Tokyo, Japan) and sequenced with a genetic analyzer 3130xl (Applied Biosystems, Foster City, CA). DNA encoding residues 18–251 were amplified with a primer set of F2 (5′-AGGTAATACACCATGACTAAAGGAAAAAAACTGGT-3′) and R2 (5′-CACCTCCACCGGATCCTTGATACACCTTAATATAGTC), and PCR was conducted as described above.

The ULam111 gene was cloned into a modified pCold I vector (Takara Bio, Shiga, Japan) as previously described^27^ between the *Nco* I and *Bam*H I sites using an In-fusion HD cloning kit (Clontech, Mountain View, CA). The expressed ULam111 protein was fused to a modified octahistidine (His_8_) tag (HHHHHHHH) at the C-terminus connected by a linker of GSGGGGGGGG. The plasmid vector was transformed into *Escherichia coli* Rosetta gami 2(DE3) (Merck Millipore, Billerica, MA) for protein expression.

The *E*. *coli* transformants were incubated at 37 °C until the optimal density at 600 nm (OD_600_) reached 0.6–0.8. Isopropyl β-D-1-thiogalactopyranoside (IPTG) was added at a final concentration of 0.5 mM, and the culture was further incubated for 16 h at 15 °C. After harvesting, the cells were disrupted by sonication in the resuspending buffer containing 50 mM sodium phosphate (pH 7.8), 10 mM imidazole, 100 mM NaCl and 1% Triton X-100. Cell debris was removed by centrifugation at 40,000 *g*. ULam111 was trapped on TALON resin (Clontech), which is a key purification step because ULam111 was partly trapped on Ni-NTA Superflow resin (QIAGEN, Hilden, Germany) in the resuspending buffer. After washing with buffer 1 [50 mM sodium phosphate (pH 7.8), 20 mM imidazole, 300 mM NaCl] and buffer 2 [20 mM Tris-HCl (pH 7.5), 100 mM NaCl], the His_8_-tagged protein was eluted with elution buffer [20 mM Tris-HCl (pH 7.5), 300 mM imidazole, 300 mM NaCl]. The eluted solution was dialyzed in buffer 2 and further purified using a Resource Q (GE Healthcare, Chicago, IL) column. The fractions containing purified ULam111 were dialyzed against buffer 2 and concentrated to 10 mg ml^−1^ for crystallization using a Vivaspin-20 (10 000 MWCO).

To obtain the selenomethionine-labeled ULam111 (ULam111^SeMet^), cells were transferred into M9 medium supplemented with 50 mg ml^−1^ selenomethionine (SeMet), when the OD_600_ reached 0.5^33, 34^. The expression and purification of ULam111^SeMet^ were the same as the native protein described above.

Site-directed mutagenesis was performed by PCR with a QuikChange kit (Stratagene, La Jolla, CA) and pCold-ULam111 plasmid as a template. The mutations were confirmed by DNA sequencing. ULam111 mutants were expressed and purified using the method described above for wild-type ULam111.

### Crystallization and data collection

Crystallization experiments were performed using the sitting-drop vapor diffusion method at 20 °C. Crystallization drops were prepared by mixing 1 μL of the protein solution with 1 μL of a variety of reservoir solutions. Crystals of native ULam111 were obtained with a reservoir solution containing 0.1 M sodium citrate (pH 5.6), 30% (w/v) polyethylene glycol (PEG) 4000 and 0.2 M ammonium acetate. Crystals of ULam111^SeMet^ were obtained with a reservoir solution containing 0.1 M sodium acetate (pH 4.6), 30% (w/v) polyethylene glycol monomethyl ether (PEG MME 2000), and 0.2 M ammonium sulfate.

The X-ray diffraction data for the native ULam111 and ULam111^SeMet^ crystals were collected on the BL-5A beamline at the Photon Factory (Tsukuba, Japan). X-ray diffraction data were collected at a resolution of 1.60 Å for native ULam111 and a resolution of 2.2 Å for ULam111^SeMet^. All diffraction data were indexed, integrated, and scaled with the XDS program^35^. The data collection statistics are summarized in Table 1.

### Structural determination

The initial phase of ULam111^SeMet^ was obtained using single anomalous dispersion (SAD)^36^. After selenium atom search and phase calculations with PHENIX AutoSol Wizard in the PHENIX program suite^37^, model building was automatically carried out with PHENIX AutoBuild Wizard^37^. Manual rebuilding and refinement of ULam111^SeMet^ were performed with COOT^38^ and PHENIX.REFINE^35^. The structure of native ULam111 was determined by the molecular replacement method using the MOLREP program^39, 40^ with the ULam111^SeMet^ structure as the initial model. Manual rebuilding and refinement of native ULam111 was performed with COOT^36^ and PHENIX.REFINE^37^, respectively. The geometry of the final structure was evaluated with the program Rampage^41^. The coordinates of ULam111 have been deposited into the Protein Data Bank (PDB) with the accession number (5WUT).

### Structural analysis

Structural analysis was carried out using a set of programs: Dali^29^ was used for the search of similar structures from the database^42^, DaliLite^43^ was used for the superposition of molecules, ESpript^44^ was used for the preparation of alignment figures, and Pymol (http://pymol.sourceforge.net/) for the depiction of structures.

We failed to obtain cocrystals of ULam111 and laminarin. Two types of tetrasaccharides (β-1,3-linkages and β-1,3-1,4-linkages, respectively) were modeled on the ULam111 structure based on the superposition of β-1,3-glucanase ZgLamA from *Zobellia galactanivorans* (PDB code: 4BOW; sequence identity, 34%; preferable β-1,3-linkages) and β-1,3-1,4-glucanase H(A16-M) (PDB code: 1U0A; sequence identity, 17%; preferable β-1,3-1,4-linkages). Gluconolactone was modeled on the superposition of TmLam from *Thermotoga maritima* (PDB code: 3AZX).

### Activity assay

Measurements of Ulam111 activity were performed using the ferricyanide reducing sugar assay^45^. The reaction conditions were as follows: 0.3% (w/v) laminarin, 10 mM NaPi (pH 6.0), 100 mM NaCl, 0.1 mg ml^−1^ BSA, and 0.1 μg ml^−1^ ULam111 at 30 °C. One unit (1 U) was defined as the amount of enzyme required to liberate 1 μmol of reducing sugar per minute. Data are presented as the mean ± S.D. from three independent experiments. The substrate specificity was assessed according to the oligosaccharide degradation of laminarin A (β-1,3-1,6-linkages) from *Laminaria digitata* (Sigma-Aldrich, St. Louis, MO), laminarin B (β-1,3-1,6-linkages) from *Eisenia bicyclis* (Tokyo Chemical Industry, Tokyo, Japan), glucan (β-1,3-1,6-linkages) from *Flammulina velutipes* ^46^, curdlan (β-1,3-linkages) from *Alcaligenes faecalis* (Wako Pure Chemical Industries, Osaka, Japan), and lichenin (β-1,3-1,4-linkages) from *Cetraria islandica* using thin-layer chromatography (TLC) with a TLC-60 plate (Merck, Darmstadt, Germany). The reaction proceeded at 30 °C for 24 h and the reaction mixture was composed of 0.5% (m/v) substrates, 10 mM NaPi (pH 6.0), 100 mM NaCl, 0.1 mg ml^−1^ BSA, and 0.02 mg ml^−1^ ULam111. Samples were developed with a solvent mixture consisting of ethyl acetate, acetic acid, and water [2:2:1 (v:v:v)] and visualized by spraying 10% (v/v) sulfuric acid in ethanol followed by heating at 130 °C for 10 min. The thermostability of ULam111 was assessed by measuring the activity after heat treatment at 15–60 °C for 15 min in the reaction mixture; 1.0 mM EDTA or CaCl_2_ was added prior to thermostability measurements.

Kinetic studies of ULam111 wild-type or F212W were performed in a solution containing 10 mM NaPi (pH 6.0), 100 mM NaCl, 0.1 mg ml^−1^ BSA, 1.0 mM CaCl_2_, 1 μg ml^−1^ wild-type or F212W enzymes, and various concentrations of substrates at 30 °C. Kinetic parameters, *K*_m_, *V*_max_, and *k*_cat_ were calculated using the Michaelis-Menten equation with GraphPad Prism 7.0 (GraphPad software, La Jolla, CA) by employing nonlinear regression.

### Data availability

Protein Data Bank (PDB): Coordinates and structure factors for the wild-type ULam111 have been deposited in the RCSB Protein Data Bank under the accession codes (5WUT).

## Electronic supplementary material

supplementary information
