# Author Correction: Laminarinase from *Flavobacterium* sp. reveals the structural basis of thermostability and substrate specificity

**DOI:** 10.1038/s41598-018-27007-x

**Published:** 2018-06-06

**Authors:** Hui-Min Qin, Takuya Miyakawa, Akira Inoue, Akira Nakamura, Ryuji Nishiyama, Takao Ojima, Masaru Tanokura

**Affiliations:** 1 Laboratory of Basic Science on Healthy Longevity, Department of Applied Biological Chemistry, Graduate School of Agricultural and Life Sciences, The University of Tokyo, 1-1-1 Yayoi, Bunkyo-ku, Tokyo, 113-8657 Japan; 2 College of Biotechnology, Tianjin University of Science and Technology, No. 29, 13th Avenue, Tianjin, 300457 China; 3 Laboratory of Marine Biotechnology and Microbiology, Graduate School of Fisheries Sciences, Hokkaido University, 3-1-1 Minato-cho, Hakodate, 041-8611 Japan

Correction to: *Scientific Reports* 10.1038/s41598-017-11542-0, published online 12 September 2017

This Article contains a typographical error in Table 1. In the ‘Data Collection’ column, “R _meas_” is incorrectly listed as “R _sym_”.

