# Supplementary material for: Laminarinase from Flavobacterium sp. reveals the structural basis of thermostability and substrate specificity
Source: Sci Rep. 2017 Sep 12;7:11425. doi: 10.1038/s41598-017-11542-0 (PMC5595797; doi:10.1038/s41598-017-11542-0)
Supplement: Supplementary file 1 — supplementary information [file 41598_2017_11542_MOESM1_ESM.pdf]

Supplementary Information for

Laminarinase from *Flavobacterium* sp. reveals the structural basis of thermostability and substrate specificity

Hui-Min Qin[1,2], Takuya Miyakawa[1], Akira Inoue[3], Akira Nakamura[1], Ryuji Nishiyama[3], Takao Ojima[3], Masaru Tanokura[1,2]*

Affiliations:

[1]Department of Applied Biological Chemistry, Graduate School of Agricultural and Life Sciences, The University of Tokyo, 1-1-1 Yayoi, Bunkyo-ku, Tokyo 113-8657, Japan.

[2]College of Biotechnology, Tianjin University of Science and Technology, No. 29, 13th Avenue, Tianjin, 300457, China.

[3]Laboratory of Marine Biotechnology and Microbiology, Graduate School of Fisheries Sciences, Hokkaido University, 3-1-1 Minato-cho, Hakodate, 041-8611, Japan.

*Corresponding author. E-mail: amtanok@mail.ecc.u-tokyo.ac.jp

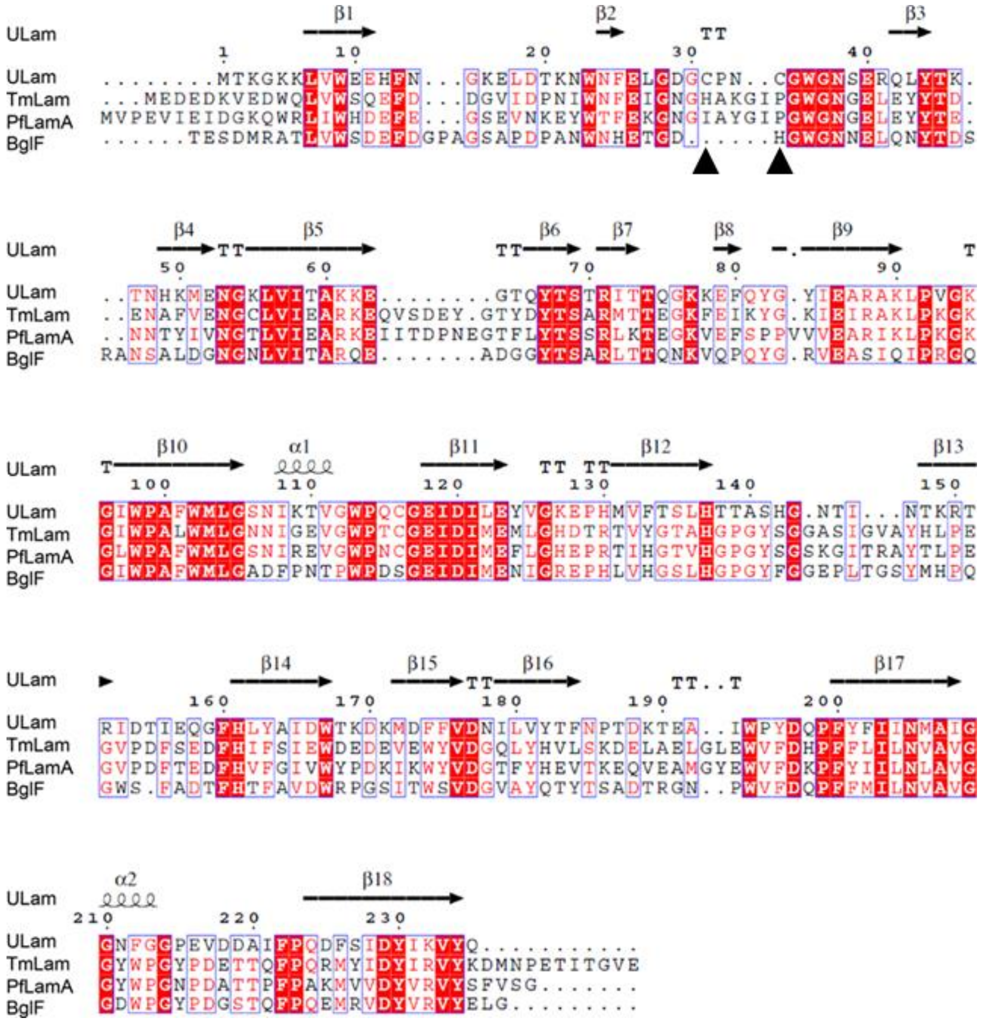

Figure S1. Amino acid sequence alignment of ULam111 with TmLam, PfLamA, and BglF. The secondary structure elements of ULam111 are depicted at the top of the alignment.

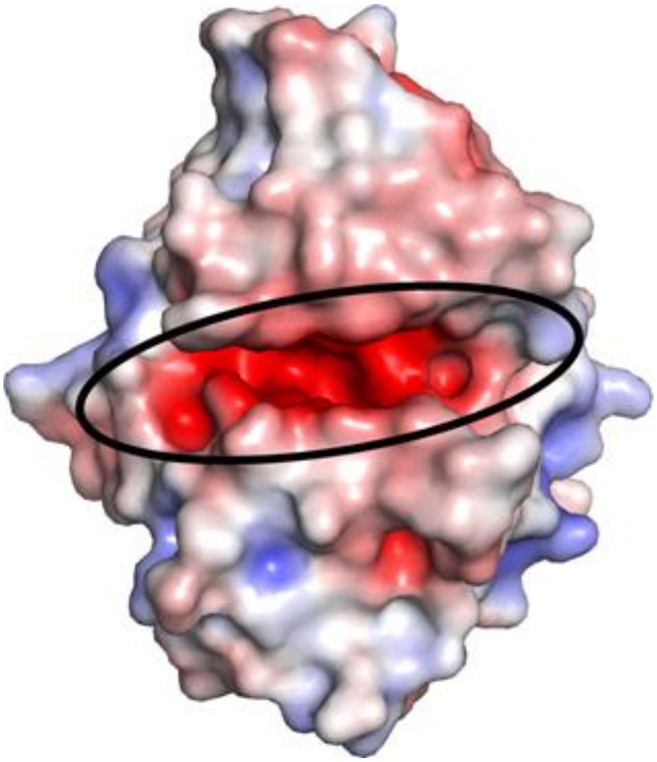

Figure S2. Electrostatic surface potential, displayed in blue for positive ($5kTe^{-1}$), red for negative ($-5kTe^{-1}$) and white for neutral. The substrate-binding concavity of ULam111 is indicated in the black circle.

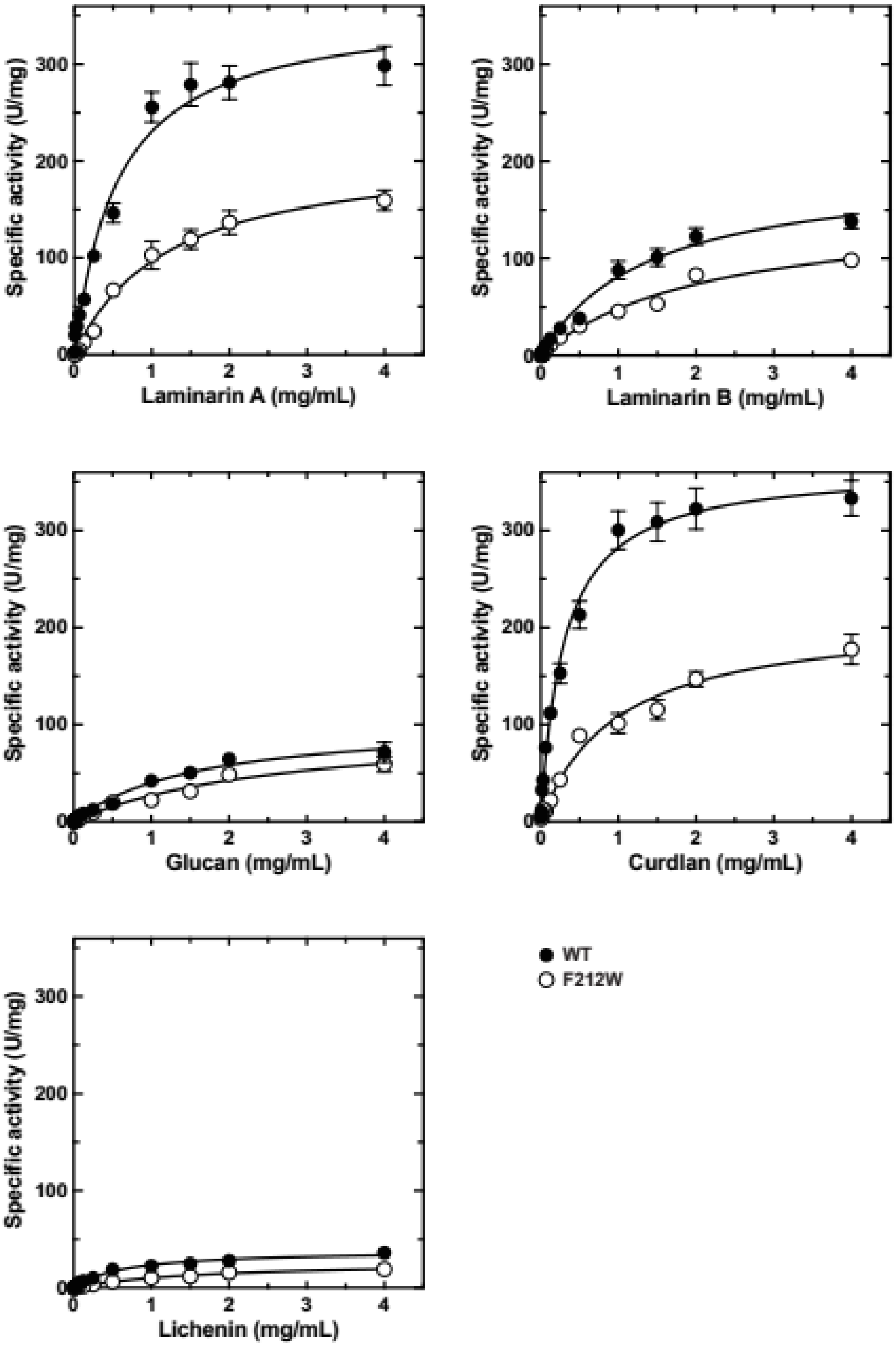

Figure S3. The fitted curves of specific activity versus substrate concentration plots of ULam111 (solid circle) and F212W mutant (hollow circle). All assays were repeated three times, and the data are shown as mean ± S.D.

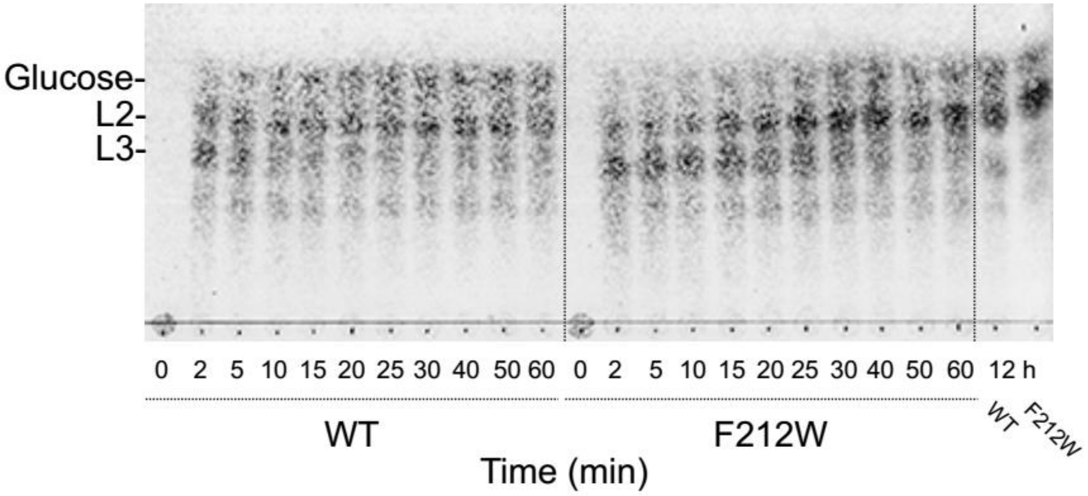

Figure S4. Hydrolysis of laminarin by ULam111 wild-type and F212W mutant was monitored by TLC. L2, laminaribiose; L3, laminaritriose.

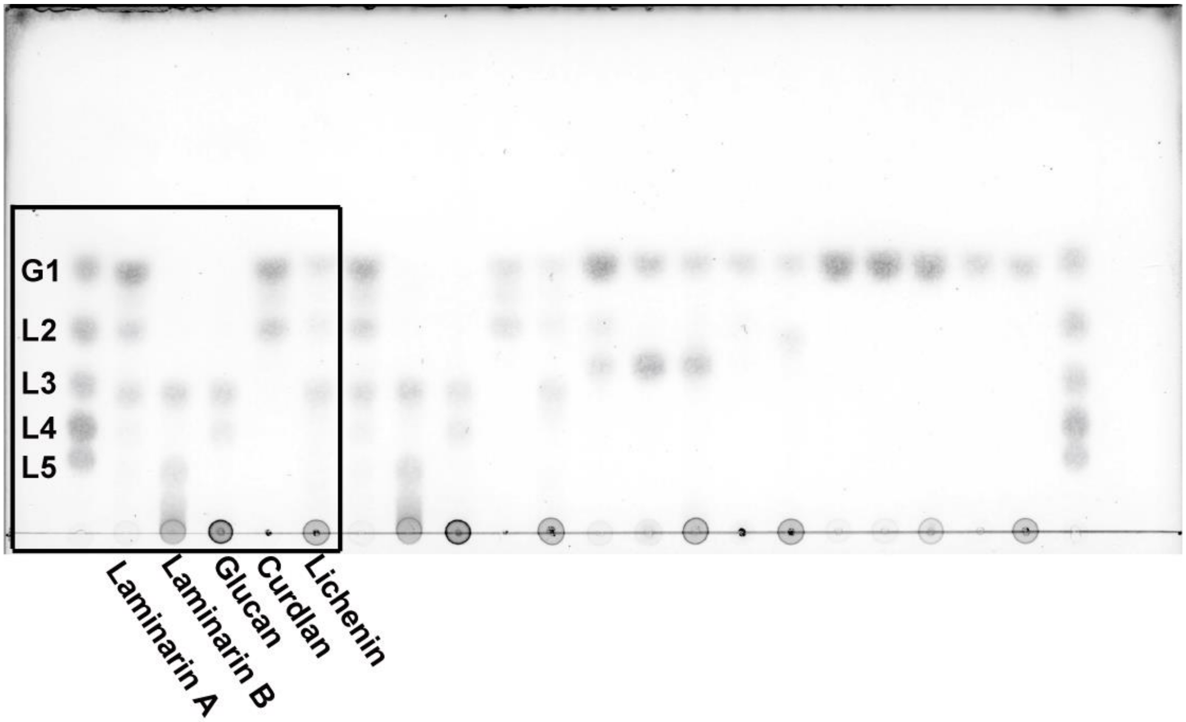

Figure S5. The original image of figure 5. Hydrolysis of laminarin by ULam111 wild-type was monitored by TLC.
